# Metacognition: computation, biology and function

**DOI:** 10.1098/rstb.2012.0021

**Published:** 2012-05-19

**Authors:** Stephen M. Fleming, Raymond J. Dolan, Christopher D. Frith

**Affiliations:** 1 Center for Neural Science, New York University, 6 Washington Place, New York, NY 10003, USA; 2 Wellcome Trust Centre for Neuroimaging, University College London, 12 Queen Square, London WC1N 3BG, UK; 3 Center of Functionally Integrative Neuroscience, Aarhus University, 8000 Aarhus, Denmark; 4 All Souls College, University of Oxford, High Street, Oxford OX1 4AL, UK

**Keywords:** metacognition, neurobiology, computational modelling, consciousness

## Abstract

Many complex systems maintain a self-referential check and balance. In animals, such reflective monitoring and control processes have been grouped under the rubric of metacognition. In this introductory article to a Theme Issue on metacognition, we review recent and rapidly progressing developments from neuroscience, cognitive psychology, computer science and philosophy of mind. While each of these areas is represented in detail by individual contributions to the volume, we take this opportunity to draw links between disciplines, and highlight areas where further integration is needed. Specifically, we cover the definition, measurement, neurobiology and possible functions of metacognition, and assess the relationship between metacognition and consciousness. We propose a framework in which level of representation, order of behaviour and access consciousness are orthogonal dimensions of the conceptual landscape.

## 1. Introduction

Many complex systems maintain a self-referential check and balance. In a democracy, voters may be consulted not only on which politician should hold office, but also about the political structure itself, as when a referendum is held on the voting system. This consultation may lead, in effect, to the political structure changing itself—an example of ‘metapolitics’. The prefix ‘meta’ derives from the Greek for ‘beyond’ or ‘with’ to indicate a concept that abstracts from another concept, often (but not always) with a self-referential flavour. An individual similarly has the ability to reflect upon, comment about, and report a variety of mental states. Metacognition—cognition about cognition—thus forms an umbrella term under which to group such mental phenomena. While cognitive science has begun to outline the workings of phenomena such as vision and memory (albeit with much work left to be done), a computational and a neural understanding of metacognition remain at an early stage.

The articles collected in this issue begin to tackle these problems by drawing on multiple levels of analysis, from single-neuron recordings to social psychology. We hope that this cross section will lead to further integration between disciplines, and provide a ‘big picture’ of how different theoretical constructs and empirical findings within the metacognition literature might relate to one another. To the extent that an account of the neural basis of metacognition informs an understanding of the representational structure of mind in humans and other animals (see §6), this research project can guide (and be guided by) parallel developments in philosophy of mind.

In this introductory article, we provide an overview of the field and direct the reader to articles within the volume that may be of particular interest. While we pursue a balanced summary of contributors’ views, we also aim for synthesis.

Until relatively recently, the empirical study of reports about cognition was limited to the domain of memory, where a theoretical framework and subtle empirical tools were developed to study the relationship between cognition about cognition, and cognition itself (the ‘meta’ and ‘object’ levels, respectively [1]). A subjectively rich example is the ‘tip of the tongue’ (TOT) state: if I ask you to recall Elton John's real name, you might have a strong feeling that you know the answer without being able to recall it. In memory research, metacognition was invoked to account for the ability of subjects to strategically control their learning, which Tulving & Madigan [2] termed one of the ‘truly unique characteristics of human memory; its knowledge of its own knowledge’. For example, a student who recognizes a lack of fluency in an upcoming exam topic might be motivated to take a trip to the library to brush up on the relevant material. Much progress has been made in understanding the psychology of metamemory and its putative role in the control of behaviour, developments comprehensively covered in recent authoritative collections [3,4]. This issue does not specifically focus upon metamemory, although many contributions inevitably draw on this literature. Instead, metacognition is characterized as operating in several domains, freeing it from the constraints of particular paradigms. As we shall see, this broader framework may have distinct advantages for computational accounts of metacognitive processes (which may be particularly tractable in areas such as decision-making), and for establishing the presence or absence of metacognition in non-human animals.

## 2. Definition and epistemological status

**Figure 1.**
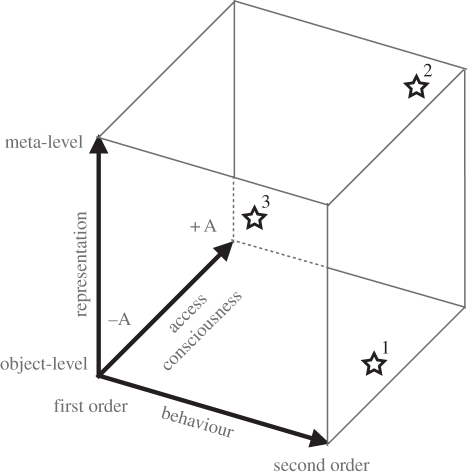
A three-dimensional map of the conceptual landscape in metacognition research. There are potential dissociations between levels of representation, orders of behaviour and access consciousness. Stars indicate our best guess of where some of the theories and measures outlined in this overview are situated in this landscape. 1, ‘direct translation’ models of decision confidence [7]; 2, representational–redescription theory of consciousness [8]; 3, first-order responses to a suprathreshold stimulus. It remains an empirical question as to the degree of independence between the dimensions sketched here.

Metacognition is a broad term, and often interpreted differently by different researchers. As a first step, it is crucial to separate the empirical definition of metacognition from its epistemological status as a meta-level representation of an object-level cognition. Empirically, metacognition is often operationalized as ‘behaviour about behaviour’ rather than ‘cognition about cognition’ ([5]; see table 1 in Fleming & Dolan [6]). Here, we define second-order behaviours as decisions contingent on other behavioural outputs (that either have occurred or will occur). Consider a visual detection task. Following a first-order response as to whether the stimulus is present or absent, a confidence judgement in one's response being correct is second-order with respect to the previous decision. This does not necessarily entail that the second-order judgement requires a meta-level representation of the object-level decision; it could instead be accomplished via object-level representations, for example, by basing confidence on information about the stimulus. Alternatively, the confidence judgement could be based on a meta-representation of the decision and subsequent response. This creates an initial division of the theoretical landscape, with two orthogonal dimensions—those of *level of representation* and *order of behaviour* (figure 1).

Often, researchers restrict the definition of metacognition to the kind of second-order behaviour available for subjective report [6]. In many of these cases, it seems intuitive that the report is capturing an aspect of cognition that is secondary to the cognitive process itself. Take the case of blindsight [9]: in some patients with lesions to primary visual cortex, visuomotor performance when responding to targets in the ‘blind’ field may be well above chance, yet the patient reports not seeing anything. This is a case where first-order (visuomotor) performance is high, but awareness is absent. Yet, the reliance on subjective reports to index metacognition precludes the ascription of metacognition to non-human animals and non-verbal infants, and may prematurely equate metacognition with consciousness (see §6). In contrast, non-verbal behavioural measures do not suffer from these drawbacks. Smith *et al.* [10] review a large body of work in non-human animals using the ‘uncertain-option’ paradigm. In these studies, some animals adaptively use an ‘uncertain-option’ response to opt-out of difficult perceptual decisions. Such behaviour alone is first-order, and Smith *et al.* consider several objections to a metacognitive characterization of the uncertain-option task. The authors point out that humans report feelings of uncertainty when making similar responses, and argue we should not hold back from a metacognitive explanation of behaviour in the case of non-verbal responses by non-human animals.

Kepecs & Mainen [7] agree that we should not set the bar higher for non-human animals than humans, and point out that it is often taken for granted that judgements of uncertainty in humans involve metacognition. They suggest that things become clearer when a non-verbal measure is elicited separately from the object-level cognition, helping to rule out alternative explanations of the data. Kepecs & Mainen [7] present data from a novel measure that satisfies these constraints: how long is an animal willing to wait for a reward after completing a decision? Importantly, such a measurement strategy permits an analysis of metacognitive accuracy—the relationship, on a trial-by-trial basis, between first-order performance and second-order behaviour (waiting time)—that is contingent on performance [6]. For example, if my willingness to wait for a reward increases in tandem with the probability of being correct, then, in this case, I can be ascribed a high degree of metacognitive accuracy.

Despite this focus on second-order behaviour, it may be premature to conclude that such behaviours require meta-representation (figure 1). For example, Kepecs & Mainen [7] outline a computational model in which the strength of evidence supporting a response option is used to guide both choice and reports of confidence (see §4). This is an example of the ‘direct-translation’ class of models [11], where the same information governs both first- and second-order behaviour. In contrast, Yeung & Summerfield [12] review a class of models in which post-decision processing contributes to metacognitive report. Such models can accommodate dissociations between the object-level and the meta-level, such as when brain damage selectively affects metacognitive accuracy, but not task performance [8,9,13]. However, and this is a crucial point, whether the second-order behaviours often used to index metacognition can be explicable in non-metarepresentational terms remains an empirical question. We might find that an object-level account is sufficient to explain second-order behaviour in some circumstances, but not others. On the other hand, evidence from human neuropsychology that first- and second-order behavioural performances are dissociable suggests that at least some degree of separate representation will be required to account for second-order behaviours [6,9,14].

## 3. Types of metacognition

A related issue is that of the *referent* of metacognitive judgements. Overgaard & Sandberg [15] compare and contrast types of metacognitive report that have been used in the literature on perceptual decision-making. In a typical task, participants make a visual judgement (such as indicating whether a stimulus is present or absent) and then provide a second-order report about this judgement. Overgaard and Sandberg contrast judgements about the *process* of cognition (confidence in having been correct) with introspective judgements about the visual *content* (such as stimulus visibility). Relatedly, Timmermans *et al.* [8] suggest that neural network architectures for metacognition should represent ‘not only the content but also the accuracy of … knowledge’. While reports about content and process are often correlated (i.e. visibility ratings tend to covary with confidence ratings), with careful analyses, report-specific features begin to emerge [15]. While this debate is currently most developed in the visual decision literature, one can imagine a similar content versus process distinction being made in domains such as memory (compare feeling-of-knowing and judgements of learning). Further, Overgaard & Sandberg [15] regard introspection as a special case: ‘ … metacognition is functionally defined … [whereas] introspection can only be about a specifically conscious state’. We will return to the problem of relating metacognition to consciousness in §6.

Bahrami *et al.* [16] also examine the role of report type but with a focus on social context. Pairs of subjects carried out visual contrast discriminations, and, in cases of disagreement, were allowed to confer to come to a joint decision. A previous study found that when the subject pairs had similar perceptual sensitivities, joint performance was better than either individual alone. In contrast, when their sensitivities markedly differed, collaboration was detrimental [17]. In the current issue, follow-up experiments are reported in which report type—verbal or non-verbal—was systematically manipulated [16]. Intriguingly, the harmful effects of collaboration for mismatched individuals are alleviated when a non-verbal report schema is used (a visual analogue scale for confidence). In contrast, when group members had similar sensitivities then best performance was achieved through direct verbal communication. These results suggest that different aspects of metacognition play different roles in social communication.

Metacognitive judgements may differ in the level to which they refer. Metcalfe *et al.* [18] (see also [19]) make a distinction between three levels of metacognitive judgements: *anoetic*, concerning objects in the world; *noetic*, concerning mental representations; and *autonoetic*, in which the referent includes the self. We note that the distinction between *anoetic* and *noetic* may relate to the distinction between judgements of *content* and *process* raised above. Metcalfe *et al.* [18] operationalize *noetic* metacognition as judgements of performance, and *autonoetic* metacognition as judgements of agency. They demonstrate that, compared with controls, patients with schizophrenia manifest aberrant judgements of agency, but intact judgements of performance in a dynamic motor task. In §5 below, we consider how cognitive neuroscience may shed light on domain-general versus domain-specific accounts of metacognition.

## 4. Computation: confidence as a test case

Second-order judgements are often concerned with the relative certainty or uncertainty of a first-order mental state. Consider a feeling-of-knowing judgement as tapping the certainty of a memory, or a visibility judgement as tapping the certainty of perception. More recently, neuroscientists have focused on confidence, or its complement, uncertainty, as a quantity that is tractable both for computational modelling and neural analysis [20,21]. Further, a computational definition of confidence can formalize intuition, and potentially unify several semantic concepts within a common framework [7].

Both Kepecs & Mainen [7] and Yeung & Summerfield [12] review recent computational models of decision confidence. Kepecs & Mainen [7] propose a signal detection theoretic model of confidence, such that confidence is equated with the evidence supporting one choice over another. Such a model can be extended to the dynamic case, by postulating accumulation of evidence over time [22], and instantiated in neural networks [23]. In contrast, Yeung & Summerfield [12] highlight a central role for post-decisional processing in the construction of metacognitive estimates of confidence. They draw together hitherto disparate literatures on error-monitoring and decision confidence, and propose that these represent special cases of a more general class of second-order decisions. In particular, they point out that fluctuations in graded judgements of confidence are often studied by changing the *quality* of evidence (such as visual contrast), whereas error-monitoring research focuses on changing the *quantity* of evidence (such as when participants are placed under time pressure). Both variables can be understood in the context of the drift-diffusion model and its post-decisional extensions that account for changes of mind and error correction. Despite this elegant synthesis, the authors argue that in the real world, sensorimotor interaction unfolds continuously, and the concept of a ‘post-decision’ metacognitive judgement may be less applicable. Instead, they propose that the *precision* of a neural representation (the reciprocal of its variance) at any given moment in time is used to inform judgements of confidence about the represented mental property.

Intriguingly, other contributions to the issue also make use of the concept of confidence-as-precision. Bahrami *et al*. [16] model confidence as the ratio of the mean of the sensory estimate to its standard deviation or, equivalently, to the product of the mean and its precision. Ko & Lau [9] propose that criteria for report in a visual detection task are set based on knowledge of one's signal-to-noise statistics (see also Lau [24]). These ideas are potentially consistent with recent neural coding models, which propose that patterns of firing in cortex encode parameters of the probability distribution that is the result of probabilistic inference, i.e. both its mean and covariance [25]. Empirical work is now required to compare and contrast different computational hypotheses regarding decision confidence.

A second problem for computational models of metacognition is how confidence information is ‘read out’ for use in second-order behaviours. Timmermans *et al*. [8] demonstrate that this type of computation can be achieved via a two-layer neural network architecture, in which the second-order network receives information about the performance of the first-order network and uses this information to generate reports of confidence. Such a model has the attractive property of accounting for dissociations between first-order and second-order decision performance. One interesting possibility is that higher-order brain areas in prefrontal cortex (PFC) explicitly represent the precision of object-level representations, thereby linking Yeung & Summerfield's account of confidence with Timmermans *et al.*'s two-layer architecture. Kepecs & Mainen [7] note that this read-out process must itself be calibrated, perhaps, requiring slow learning over time; Timmermans *et al.* [8] shed light on this issue by analysing their neural network's metacognitive performance during the training phase, demonstrating gradual changes in metacognitive sensitivity and calibration (as assessed via type 2 signal detection analysis).

There is a rich literature on the computation and usage of uncertainty to optimize decision-making  [25,26], but one that often remains separate from the literature on metacognition. We suggest that a profitable avenue for future research is the degree to which such models of behaviour account for metacognitive reports, situating metacognition more precisely in relation to other aspects of behaviour (see Maniscalco & Lau [27], for a related approach).

## 5. Neurobiological substrates of metacognition

Early accounts of the neural substrates of metacognition focused on patients with lesions to the PFC and impaired metamemory (see Shimamura [28], for a review). These dissociations were interpreted in the context of the PFC implementing the meta-level of Nelson & Narens [1], or perhaps the second-order network in more recent computational accounts [8,23]. Fleming & Dolan [6] review recent studies that harness a variety of methods in cognitive neuroscience to draw relationships between brain structure and function and metacognitive capacity. They note that a crucial aspect of this research programme is the independent measurement of both task performance and second-order report, as inter-dependencies between these levels otherwise render it difficult to dissociate a neural correlate of one from the other (see also [29]). Studies employing such methods reveal distinct neural substrates for prospective and retrospective second-order judgements, and suggest the existence of rich second-order corrective behaviours accounted for by post-decisional processing [6,12]. Note that here, again, evidence for a dissociable representation governing second-order behaviour does not necessarily entail it being a metarepresetation, and further work is needed to understand the interactions between putative prefrontal representations controlling second-order behaviour and object-level representations underlying first-order behaviour.

The development of measures of second-order behaviour in non-human animals [10] has led to the possibility of direct recordings from single neurons during second-order judgements. Previous studies have relied on an object-level framework, where confidence is equated with the information that governs the choice process [30–32]. As noted above, this framework potentially confounds neural activity representing confidence from that representing the graded value of one decision option over the other. However, the development of paradigms in which confidence and choice are measured independently on each trial suggests that neural recordings relating task performance to confidence in non-human animals are likely to be documented in the near future [7,33].

A related perspective on the neural basis of metacognition is provided by examining neuropsychiatric cases in which metacognition is systematically impaired. As noted in §3, these studies often tap into a self-related component of metacognition: ‘insight’ into a characteristic of one's behaviour or illness. David *et al.* [14] review a series of studies examining metacognitive function in patients with Alzheimer's disease, brain injury or schizophrenia, and relate these measures to assessment of clinical insight. Together, their work suggests fractionation between different types of insight, such that insight into one aspect of pathology does not necessarily predict insight into another. Furthermore, they show that the activity of anterior medial PFC is increased during self-reflection in controls, but not in schizophrenic patients. They suggest that medial PFC dysfunction may mediate metacognitive deficits in psychopathology.

## 6. Relationship to conscious awareness

Defining consciousness is notoriously tricky, even more so than defining metacognition. Block draws a distinction between phenomenal consciousness—the ‘what it is like’ of experience—and access consciousness, which includes availability to cognitive processing, the capacity to report, act and so forth [34]. It is not immediately clear how second-order behaviours fit into this framework. In particular, access consciousness is present in a large number of first-order cases, such as when one responds to or classifies a stimulus. Conversely, Fleming and Dolan [6] note that error-related (second-order) adjustments in behaviour can occur in the absence of subjective reports (e.g. [35]; see also [12]). Thus, *access consciousness* may represent a third orthogonal dimension of our theoretical landscape (figure 1). Nevertheless, several authors have suggested intimate links between metacognition and conscious awareness [5,36,37], and a widespread use of second-order behavioural measures as empirical indices of awareness makes it important to critically appraise this link.

On the empirical side, Overgaard & Sandberg [15] draw a distinction between introspection and metacognition, the former being a special case, where participants are asked to comment directly on conscious experience. Furthermore, they argue that both introspection and metacognition are dissociable from report, motivating the use of measures such as type 2 signal detection theory that discount response bias to assess introspective access. An interesting case study is that of blindsight. Here, as noted above, metacognitive measures dissociate from above-chance performance, and do so in a way that is difficult to account for in a traditional signal detection framework. Ko & Lau [9] propose that the lack of cognitive access in blindsight can be explained by a failure of criterion setting based on inaccurate meta-level knowledge of internal probability distributions following the lesion. Such a failure to update subjective criteria is supported by a computational model in which a rapid decrease in visual sensitivity is accompanied by suboptimal criterion setting. This perspective suggests that metacognition may *enable* reliable cognitive access (through accurate criterion setting over time), but does not necessarily predict whether a given trial will be reported as ‘aware’ or ‘unaware’.

On the theoretical side, Timmermans *et al.* [8] propose that consciousness is a representational–redescription process, with a hierarchy of predictive loops gradually learning to re-represent first-order mental states. As such, consciousness is intimately bound to meta-representation, and they suggest that meta-representational capacity can be assessed by the relationship between metacognitive reports and behaviour. As noted above, a deflationary explanation of some types of metacognitive report that does not appeal to meta-representation may be possible, and it will important to consider this possibility on a case-by-case basis [38].

Rosenthal has previously proposed an influential higher-order thought (HOT) theory of consciousness in which mental states are conscious in virtue of a metarepresentational state—an HOT—about them [39]. Rosenthal, like Timmermans *et al*. [8], notes that being aware of a mental state appears to share common ground with accounts of metacognition. Indeed, the link between introspective reports and PFC function has recently been deployed in support of HOT theory [40]. However, Rosenthal [41] argues that although consciousness and metacognition involve higher-order capacity, they have little more in common. In particular, Rosenthal points out that metacognitive states, such as the tip-of-the-tongue state discussed above, do not make one aware of the mental state (the memory) it refers to, and thus are distinct from HOTs. Introspection of content [15] may be a better candidate for a link between HOT and metacognition, and Rosenthal agrees that while these reports may index HOTs, they also induce a rare third-order state, in which one is aware of one's HOT.

As this brief summary indicates, the link between metacognition and consciousness inevitably depends on one's favoured definition of each. As depicted in figure 1, access consciousness may dissociate from both second-order behaviour and meta-level representation, opening up new questions for empirical research aimed at populating this conceptual landscape. For example, it will be important to assess whether having meta-level content is necessary and sufficient for a state to be conscious, and the type of behavioural measure that will allow us to reliably identify such a meta-level state. Progress in resolving this question will undoubtedly benefit from an interaction between theorists developing accounts of consciousness and empirical researchers refining the measurement of metacognitive behaviour.

## 7. The function of metacognition

The function of metacognition has usually been construed in terms of control of behaviour and mental processes: the implication is that accurate control requires accurate monitoring [1]. However, recent attempts to distil metacognition from other potentially confounding variables have led to a seeming paradox: if, to measure metacognition, one needs first to discount the influence of first-order behaviour [27,42], then the functional benefits of metacognitive capacity would appear moot. Perhaps, instead, metacognitive capacity is not necessary for controlling simple behaviours, but becomes relevant for complex abilities such as reasoning and planning. Fletcher & Carruthers [43] critically appraise this hypothesis, presenting a deflationary account of a metacognitive system for human reasoning. They argue that reasoning can often be explained by an appeal to individual strategies acquired through individual and cultural learning. However, they also suggest that a dedicated ‘mind-reading system’ is recruited in a self-monitoring capacity to guide reasoning, an example of a system originally evolved to serve social functions playing a special role in self-directed cognition (see Carruthers [44]).

Frith has proposed that metacognitive states are useful precisely because they can be communicated to others, promoting social interaction [45]. Bahrami *et al*. [16] further suggest that communicating metacognitive confidence may act to replace explicit feedback about decision outcomes, and thus provide an ecologically relevant role for metacognition in social learning. This hypothesis is supported by empirical work demonstrating that explicit feedback is not required to reap the benefits of cooperation for decision-making. The ability to reap cooperative benefit requires reflection on performance, communication of confidence to others and comparison of one's confidence with that of the group, and metacognition presumably plays a central role in this process [46].

A natural place to look for a function of metacognition is in cases where it goes awry. David *et al.* [14] draw a link between metacognitive failure and lack of insight in psychosis, but note that the picture is far from clear: while insight is separable from primary symptomology, there is a complex relationship between measures of cognitive and clinical insight. Metcalfe *et al.* [18] point out that the neural substrates of autonoetic, or self-related, metacognition [47,48] are strikingly similar to those isolated in studies of metacognition of decision-making [6]. As lack of insight into illness is dysfunctional in terms of prognosis [49] and failure to self-medicate [50], one function of metacognition may be to promote on-going monitoring and control to the level of explicit awareness (cf. Block's ‘monitoring’ consciousness, [34]), thus allowing self-referential decisions to be made.

Finally, an intriguing idea is that metacognition is functional precisely because it enables representation of the *absence* of knowledge. Object-level representations are often concerned with presence of stimuli in the world; they rarely deal in absence (consider the failure to represent the blindspot in vision). In contrast, ‘knowing I do not know’ is a meta-level representation of the absence of object-level memory. Investigating this putative function may benefit from greater integration with work quantifying epistemic behaviour—by sampling information over time, an agent can adaptively reduce its uncertainty, achieving a balance between the additional cost of exploration and the benefit of gaining further information [51]. Interestingly, one key variable in driving further information-gathering is the current (im)precision of one's representation, providing a connection to computational models of confidence discussed in §4.

These suggestions for functional roles are not mutually exclusive. A natural direction for future research is situating our evolving mechanistic understanding of metacognition in a functional context in order to critically evaluate such hypotheses.

## 8. Conclusion

In this article, we provide an overview of the content of this Theme Issue, and draw links between different domains of enquiry. Specifically, we propose a framework in which level of representation, order of behaviour and access consciousness are orthogonal dimensions of the conceptual landscape (figure 1). We believe there is much to be gained by grounding future studies of metacognition in this framework.

Any overview is necessarily brief, and we encourage the reader to delve deeper into this Theme Issue, and to read the articles cited here. We are excited by the rapid development in this relatively young field; there is rich potential for cross-disciplinary collaborations that span several levels of analysis, from single-neuron recordings to philosophy of mind. Science is a quintessentially metacognitive activity: it continually questions itself, probing and testing the robustness of accumulated knowledge. Articles in this Theme Issue highlight the existence of some knowledge we do have, and much that we do not. By embracing just as many questions as answers, we are confident that this issue will lead to further progress in understanding the metacognitive components of mind.
